# Liming potential and characteristics of biochar produced from woody and non-woody biomass at different pyrolysis temperatures

**DOI:** 10.1038/s41598-024-61974-8

**Published:** 2024-05-20

**Authors:** Ghulam Murtaza, Muhammad Usman, Javed Iqbal, Sajjad Hyder, Farheen Solangi, Rashid Iqbal, Mohammad K. Okla, Abdullah Ahmed Al-Ghamdi, Heba H. Elsalahy, Waseem Tariq, Omar A. A. I. Al-Elwany

**Affiliations:** 1 Faculty of Environmental Science and Engineering, Kunming University of Science and Technology, Kunming, 650500 China; 2 School of Agriculture and Biology, Shanghai Jiao Tong University, 800 Dongchuan Road, Minghang District, Shanghai, 200240 People’s Republic of China; 3 Departemnt of Botany, Bacha Khan University, Charsadda, Khyber Pakhtunkhwa 24420 Pakistan; 4 Department of Botany, Government College Women University Sialkot, Sialkot, 51310 Pakistan; 5 Research Centre of Fluid Machinery Engineering and Technology, Jiangsu University, Zhenjiang, 212013 China; 6 Department of Agronomy, The Islamia University of Bahawalpur, Bahawalpur, 63100 Pakistan; 7 Department of Botany and Microbiology, College of Science, King Saud University, P.O. Box 2455, 11451 Riyadh, Saudi Arabia; 8 Leibniz Centre for Agricultural Landscape Research (ZALF), 15374 Müncheberg, Germany; 9 Department of Horticulture, Faculty of Agriculture, Fayoum University, Fayoum, 63514 Egypt

**Keywords:** Biochar, Feedstock, Pyrolysis, *Acacia nilotica* bark, Eggplant, Biochemistry, Ecology, Environmental sciences

## Abstract

Large amount of wastes are burnt or left to decompose on site or at landfills where they cause air pollution and nutrient leaching to groundwater. Waste management strategies that return these food wastes to agricultural soils recover the carbon and nutrients that would otherwise have been lost, enrich soils and improve crop productivity. The incorporation of liming materials can neutralize the protons released, hence reducing soil acidity and its adverse impacts to the soil environment, food security, and human health. Biochar derived from organic residues is becoming a source of carbon input to soil and provides multifunctional values. Biochar can be alkaline in nature, with the level of alkalinity dependent upon the feedstock and processing conditions. This study conducted a characterization of biochar derived from the pyrolysis process of eggplant and *Acacia nilotica* bark at temperatures of 300 °C and 600 °C. An analysis was conducted on the biochar kinds to determine their pH, phosphorus (P), as well as other elemental composition. The proximate analysis was conducted by the ASTM standard 1762-84, while the surface morphological features were measured using a scanning electron microscope. The biochar derived from *Acacia nilotica* bark exhibited a greater yield and higher level of fixed carbon while possessing a lower content of ash and volatile components compared to biochar derived from eggplant. The eggplant biochar exhibits a higher liming ability at 600 °C compared to the acacia nilotica bark-derived biochar. The calcium carbonate equivalent, pH, potassium (K), and phosphorus (P) levels in eggplant biochars increased as the pyrolysis temperature increased. The results suggest that biochar derived from eggplant could be a beneficial resource for storing carbon in the soil, as well as for addressing soil acidity and enhancing nutrients availability, particularly potassium and phosphorus in acidic soils.

## Introduction

Soil acidification is a major problem in agricultural production, affecting 30–40% of the world’s arable land^1^. Soil acidification is a natural phenomenon that can be expedited by specific plant species and human actions, or decelerated through meticulous management strategies. Soil acidification occurs as a result of industrial and mining activities, which generate acid through the oxidation of pyrite and the release of sulfur (S) and nitrogen (N) gas, leading to acid precipitation^1^. The acidified soil in Pakistan mainly occurs around the Indus River. Soil acidification is increasing in South Punjab due to excessive nitrogen application and the base ions elimination during harvesting, as reported by Khan et al.^2^. In agriculture, the application of natural acid-neutralizing elements such as dolomite and lime is essential to address the limitations produced by soil acidification when the soil becomes excessively acidic. Liming materials enhances the physicochemical and biological qualities of soil, consequently improving soil fertility and promoting plant growth. Applying liming materials to acidic soils not only improves soil structure but also increases the movement and accessibility of important plant nutrients through abiotic (such as phosphorus) and biotic (such as nitrogen) processes. Additionally, it helps to immobilize harmful heavy metals^3^. Various liming substances, such as agricultural lime, steel slag, and dolomite are frequently employed to neutralize soil acidity^4,5^. Biochar, a carbonaceous material formed from biomass, is often alkaline, making it suitable for use as a liming agent. For instance, according to Cui et al.^6^, the use of biochar in acidic soils led to an elevation in soil pH, which in turn resulted in favorable plant reactions^7–9^. The biochar application to acidic soils directly improves the soil's physical, chemical, and biological properties^10–12^. Biochar can be considered a soil facilitator for the movement and cycling of nutrients, the growth of plants, and its impact on soil toxicity, density, and nutrient release^13–15^. However, the material and pyrolysis temperature have the greatest influence on the effects of biochar^16^. Biochar addition to the soil increases its pH and improves nutrient availability and uptake by plants^17^. For example, biochar increases the availability of fixed phosphorous with high contents of aluminum (Al^3+^) and iron (Fe^2+^) under acidic conditions^18^, and improves the bioavailability of nitrogen (N), phosphorous (P), potassium (K), Ca^2+^, and Mg^2+^^19^. Different feedstocks have been examined for biochar production to improve soil properties, including wood-biomass, agricultural crop residues (rice straw and husk, wheat straw) as well as grasses^20^. The physio-chemical parameters of the biochar generated vary significantly depending on the feedstock utilized, which might have different impacts on soil qualities^7^.

The main factors that influence the creation of specialized biochar suitable for any scenario are the choice of raw feedstock and the temperature utilized during pyrolysis. Thus, the current study aimed to evaluate the impact of woody and non-woody feedstock types and different pyrolysis temperatures on liming potential and physicochemical properties of biochars derived from non-woody feedstock (eggplant peel) and woody feedstock (*Acacia nilotica* bark).

## Materials and methods

### Research site

The research was conducted at The Islamia University of Bahawalpur, located in the Punjab province of Pakistan at coordinates 29.3981°N and 71.6908°E.

### Characterization of eggplant peel and *Acacia nilotica* bark feedstock

Eggplant peel and *Acacia nilotica* bark were used (permissions or licenses were obtained) to make the biochar utilized in the present study. The eggplants were purchased from stores that sell eggplants. Eggplants peels used because this area is famous for eggplant production, so this is good to handle the waste of eggplant or residues after produce biochar. To expedite the drying process, the eggplants were gathered into black plastic bags, let air dry for a week in a glasshouse, and then placed into plastic bags. The bark of *Acacia nilotica* was obtained from a private forestry by-product mill situated in Khanewal, Punjab, Pakistan. It was stored for more than a month before sampling, air-dried for a period of a week, and then placed in plastic bags for up to a week before being ground. The *Acacia* bark and eggplant peel samples were pulverized to a particle size of less than 2 mm using a grinding mill machine and then placed in white plastic bags for storage. Subsequently, the particles went through oven-drying at a temperature of 80 °C for 24 h before characterization. Table 1 shows attributes of raw materials (eggplant peel and Acacia nilotica bark feedstocks).

**Table 1:** attributes of raw materials (eggplant peel and *Acacia nilotica* bark feedstocks).

Feedstocks	Eggplant peel	*Acacia nilotica* bark
Ultimate analysis		
Total H %	6.29	5.24
Total N %	1.21	0.411
Total C %	40	51.1
O %	48.9	44.5
O/C	0.1	0.07
H/C	0.013	0.08
Proximate analysis		
Fixed carbon %	16.9	30.1
Volatile matter %	77.8	69.9
Moisture content %	11.7	10.2
Ash content %	4.60	0.542
Other traits		
pH	8.09	4.30
CEC (cmolc kg^−1^)	58.1	13.4
Mg (cmolc kg^−1^)	6.41	2.31
Ca (cmolc kg^−1^)	1.09	1.31
P (mg/kg)	82.0	13.9

### Production of biochar

The biochar was generated using pyrolysis at temperatures of 300 °C and 600 °C for 2 h, following the guidelines provided by Lehmann^21^. Following the pyrolysis process, the biochar was finely crushed to a size that would allow it to pass through a filter with a 2 mm size. This was done to ensure that both biochar particles had a uniform size for further tests.

### Characterization of eggplant peel and *Acacia nilotica* bark derived biochars

#### Fixed carbon, volatile matter, moisture and ash content

The materials underwent proximate analysis according to the adopted approach stated by Wu et al.^22^. The moisture content of the milled samples was assessed by putting them to oven-drying at a temperature of 105 °C for duration of 2 h. The volatile matter, on the other hand, was evaluated by measuring the weight loss of the samples when exposed to a temperature of 950 °C for a period of 6 min in a furnace that lacked oxygen. The ash content was measured by subjecting the sample to combustion at a temperature of 750 °C for duration of 6 h in a furnace that lacked oxygen. The fixed carbon content was then computed by subtracting the ash percentage and volatile matter percentage from 100%^23^.

#### Selected physiochemical characteristics

The pH was measured in a solution of water and KCl at a ratio of 1:10 (Enders et al. 2012). The Leco Trumac (CNS) autoanalyzer equipment was used to analyze the total N and total C through dry combustion^24^. The CHN elemental analyzer was used to analyze the total hydrogen content. The total O was determined using Eq. 1 from the study conducted by Enders et al. in^25^. Calorimetric analysis was used to determine the concentration of extractable phosphorus, employing the AMBIC-2 extraction technique developed by Enaime and Lubken in^26^. The molybdenum-blue technique, as described by Murphy and Riley in^27^, was then used to examine the extracted samples. The cation exchange capacity was measured by determining the amount of NH_4_^+^ ions that remained after being washed with multiple portions of ethanol using the Thermo Scientific Gallery Discrete Autoanalyzer. The exchangeable bases were extracted via the 1M ammonium acetate procedure and their concentrations were determined via Atomic absorption spectrometry for Mg^2+^ and Ca^2+^ ions, and Atomic flame spectrometry for K^+^ ions. The calcium carbonate equivalent of biochar's liming capability was assessed using the methodology described by Bolan et al.^28^. The obtained data was used to compute the calcium carbonate equivalent using Eq. 2.

1 $$\mathrm{Total\, O }\left(\mathrm{\%}\right)=100-({\text{C}}+{\text{H}}+{\text{N}}+{\text{ASH}})$$

2 $$\mathrm{Calcium\, carbonate\, equivalent\, }(\mathrm{\%})=\frac{M\times (b-a)\times 10(-3)\times 100.09\times 100}{2\times W}$$

where 'M' represents the molarity of NaOH (in moles per liter), 'b' represents the volume (in milliliters) of NaOH used for the blank, and 'a' represents the volume (in milliliters) of NaOH used for the biochar sample. The variable 'W' represents the bulk (in grams) of biochar utilized.

#### Surface morphology

The surface properties of both biochars were examined with a Scanning Electron Microscope. The biochar samples were affixed to an adhesive C tape on an aluminum stub, coated with a gold coating for six cycles, and then observed using a gold sputtering machine.

#### Incubation in soil for determination of liming potential

Bahawalpur (29.3544°N, 71.6911°E) and Lodhran (29.5467°N, 71.6276°E) were the locations of the two soil samples used. The Bahawalpur region experiences an average annual rainfall of 227 mm, and the soil is covered by natural flora. The Lodhran region has an average annual rainfall of 205 mm, and the land is utilized for growing wheat. The soil type found at Bahawalpur was classified as Bonheim, corresponding to Luvisol, whereas the soil type in Lodhran was classified as Clovelly, corresponding to Ferralsol. Soil samples were gathered from the 0 to 20 cm depth, combined, made uniform, dried naturally, and filtered through a sieve with a pore size of less than 2mm before testing. The amount of clay was determined through the utilization of Mid-Infrared reflectance, employing the HTS-XT instrument manufactured by Bruker in Germany. Table 2 displays the key attributes of the soils. The Luvisol soil included 40% clay, had a pH _(KCl)_ value of 4.59, and a C/N ratio of 16.25. On the other hand, the Ferralsol soil had 24% clay, a pH _(KCl) _value of 3.89, and a C/N ratio of 13.34. The Luvisol exhibited higher levels of calcium (Ca) and magnesium (Mg), while having lower levels of total carbon (C) and nitrogen (N), extractable phosphorus (P), exchangeable potassium (K), and acidity compared to the Ferralsol.

**Table 2:** Key physicochemical characteristics of the applied soil.

Attributes	Luvisol	Ferralsol
pH _(KCl)_	4.59	3.89
pH _(H2O)_	5.91	4.69
Clay %	40	24
Nitrogen %	0.270	0.412
Carbon %	4.39	5.5
C/N	16.25	13.34
Bulk density g/cm^-3^	1.30	1.09
Exchangeable Magnesium (cmol_c_ kg)	2.19	0.667
Exchangeable Calcium (cmol_c_ kg)	2.18	1.01
Exchangeable Potassium (cmol_c_ kg)	0.0539	0.221
Extractable phosphorus mg/kg	2.81	17.9
Exchangeable acidity (cmol_c_ kg)	1.7	8

#### Experimental design/set-up

Two soils and four biochars made from eggplant peel and Acacia nilotica pyrolyzed at 300 °C and 600 °C were used in triplicates in the experiment, which was set up in a 2 × 4 factorial design with a completely random design. The biochar was applied as lime at the recommended rate according to the CCE, while the control group received soil that had not been treated with biochar. Based on the calculations made by Vilakazi et al.^29^ using Eq. 3, the suggested amount of lime to neutralize acidity in Luvisol soil was 5 tha^−1^, while in Ferralsol soil, it was 29 t-ha^−1^. A positive control was also provided, which was lime Calcium carbonate (CaCO_3_). The soil's moisture content at field capacity was determined by employing a pressure plate set at -33 kPa, as described by Smith and Mullins^30^. The soil (100g) and biochar were combined in 500 ml plastic containers and well-blended before adjusting the moisture level to 100% field capacity. The amount of moisture in the soil was adjusted by taking into account the weight reduction. The soils were subjected to a 10-day incubation period in a temperature-controlled chamber set at a constant temperature of 25 °C. Subsequently, the pH levels of the soils were determined and evaluated according to the methodology described by Singh et al.^31^.

3 $$Lime\, requriemnt \left(\mathrm{t ha}-1\right)=["\text{Exchangeable acidity"}-(\mathrm{total\, cations }\times {\text{PAS}}/100)]\times \mathrm{F }$$

The PAS, which represents the allowed acid saturation for the chosen crop, was set at 5% acid tolerance for this research. The factor F represents the quantity of lime needed to neutralize 1 cmolc L-1 of exchangeable acidity.

#### Analysis

The soil's content of total carbon and nitrogen, as well as the levels of extractable phosphorus and exchangeable calcium, magnesium, and potassium, were analyzed by the specifications for biochar properties. The soil pH was measured using a pH meter (Ohaus starting 2100) at a soil-to-solution ratio of 1:5 in both water and 1M KCl. The exchangeable acidity was extracted using a solution of 1 M KCl and then measured by titration with a solution of 0.1 M sodium hydroxide, using phenolphthalein as an indicator^32^.

#### Quantitative analysis

The data regarding the properties and calcium carbonate equivalents of different forms of biochar were analyzed using a two-way analysis of variance (ANOVA) in GenStat 18th edition. This analysis aimed to determine the impacts of pyrolysis temperature and feedstock type. A two-way analysis of variance (ANOVA) was conducted to evaluate the acid-neutralization capacity of different kinds of biochar in two different soils. The mean separation was conducted using the least significant difference (LSD) test at a significance level of *p* < 0.05. The Tukey–Kramer test was employed to distinguish treatment means at a significance level of *p* < 0.05 and was utilized in the description of the results.

### Ethics approval and consent to participate

This study does not include human or animal subjects.

### Statement on guidelines

All experimental studies and experimental materials involved in this research are in full compliance with relevant institutional, national and international guidelines and legislation.

## Results

### Yield of biochar, ultimate and proximate analysis

The yield of biochar reduced as the pyrolysis temperature increased for both feedstocks, as shown in Table 3. The yield of biochar was highest for eggplant, followed by *Acacia nilotica* bark having the lowest yield across all temperatures used for pyrolysis (Table 3). The moisture level of eggplant biochar was higher than that of *Acacia nilotica* bark biochar across both temperatures used for pyrolysis. However, there were no significant changes in the moisture level between the different pyrolysis temperatures. Among all the treatments, the biochar made from *Acacia nilotica* bark at 600 °C had the lowest moisture level (Table 3). Increasing the pyrolysis temperature resulted in a significant reduction (*p* < 0.05) in the amount of volatile matter and a surge in ash content and fixed carbon for both materials, as shown in Table 3. The tendencies observed were as follows: eggplant had higher ash concentrations compared to Acacia nilotica, and *Acacia nilotica* had higher fixed carbon than eggplant at both temperatures used for pyrolysis. At a temperature of 300 °C, the volatile matter content in *Acacia nilotica* biochar was notably greater than that in eggplant biochar. However, at a temperature of 600 °C, the biochar derived from *Acacia nilotica* exhibited the lowest volatile matter content. The ash level was much greater and the fixed carbon content was lower in the eggplant biochar compared to those derived from *Acacia nilotica* bark. The variations in the amount of fixed carbon were rather insignificant among the different biochars produced at 300 °C. However, at 600 °C, the fixed carbon content was notably higher for the biochar derived from *Acacia nilotica* bark.

The concentration of total carbon exhibited a consistent pattern with fixed carbon across all biochars. It increased as the pyrolysis temperature rose for both feedstocks, as shown in Table 4. The Acacia nilotica biochar exhibited higher total carbon content compared to the biochar derived from eggplant residue, at both temperatures used for pyrolysis, as shown in Table 4. The increase in pyrolysis temperature from 300 to 600 °C resulted in a substantial decrease (*p* < 0.05) in the total nitrogen content of the eggplant materials, but not in the Acacia nilotica bark. The C/N ratio of the *Acacia nilotica* bark biochar was slightly higher than that of eggplant residue at all pyrolysis temperatures, as shown in Table 4. Biochars generated at higher temperatures exhibited significantly reduced levels (*p* < 0.05) of oxygen (O), O: C, hydrogen (H), and H: C. At a temperature of 300 °C, the biochar derived from *Acacia nilotica* bark exhibited higher O content and O: C ratio compared to the biochar derived from eggplant. Conversely, at a temperature of 600 °C, the eggplant biochar had higher oxygen concentration and O/C than the *Acacia nilotica* bark biochar. There were no variations in the values of H and H/C among the biochars derived at each temperature.

### Chemical characteristics of biochar derived from woody (*Acacia nilotica* bark) and non-woody (eggplant) biomass

The pH of biochar showed a significant rise as the pyrolysis temperature increased. Biochar derived from eggplant waste had a higher pH compared to that derived from *Acacia nilotica* bark, as indicated in Table 5. At a pyrolysis temperature of 600 °C, the pH values followed the sequence of eggplant being greater than *Acacia nilotica* bark. Eggplant biochar exhibited alkaline pH values; however, the *Acacia nilotica* bark biochar generated at 300 °C displayed an acidic pH. The pH values of KCl and water varied from 4.69 to 10.2 for biochars created at 300 °C, and from 9.09 to 11.9 for biochars synthesized at 600 °C.

The concentration of extractable phosphorus increased as the pyrolysis temperature increased. Eggplant biochars had significantly greater levels (*p* < 0.05) than *Acacia nilotica* bark, at both temperatures used for pyrolysis. The ammonium acetate extractable potassium content of eggplant biochars was significantly greater (*p* < 0.05) compared to *Acacia nilotica* bark. However, there were no significant differences (*p* > 0.05) observed between the two pyrolysis temperatures (300 °C and 600 °C) as shown in Table 5. The increase of production temperature from 300 to 600 °C resulted in a reduction in the amount of magnesium (Mg) present in eggplant biochar, but there was no alteration observed in *Acacia nilotica* bark. At both pyrolysis temperatures, the trend seen in the CEC (Cation Exchange Capacity) was that the eggplant had a higher CEC than the Acacia nilotica bark. The carbon exchange capacity (CEC) of eggplant was notably reduced as the pyrolysis temperature increased from 300 to 600 °C. However, this temperature increase had no impact on the CEC of *Acacia nilotica* bark. The CCE (calcium carbonate equivalent) of eggplant biochars was significantly greater (*p* < 0.05) compared to Acacia nilotica bark. However, there were no significant variations in CCE between eggplant biochar produced at different pyrolysis temperatures.

### SEM analysis

**Figure 1:**
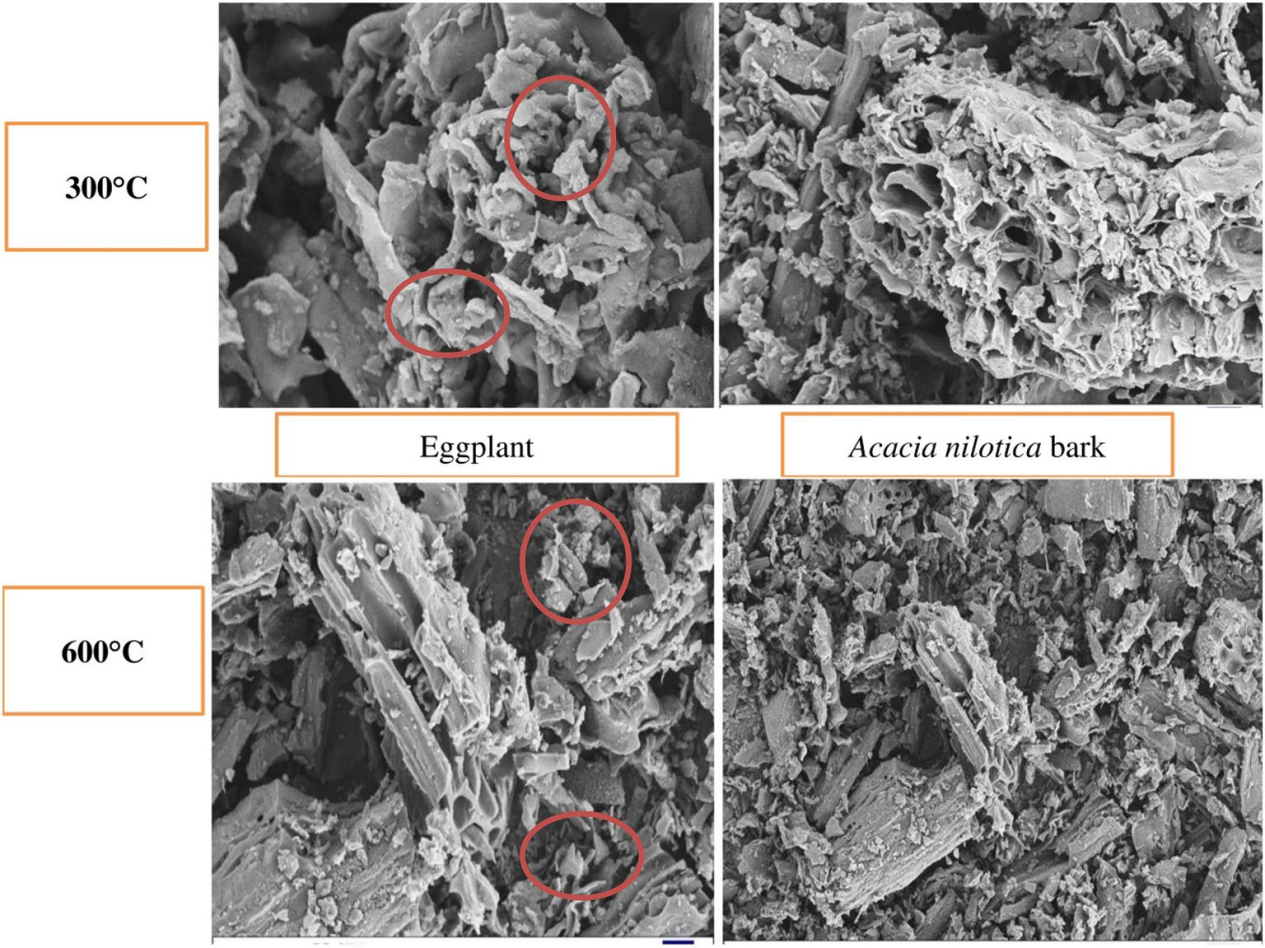
Scanning Electron Microscopy was used to compare the morphology of four biochars that were investigated at various pyrolysis temperatures.

The electron-microscope visuals revealed that the external structure of biochar was significantly altered due to pyrolysis (Fig. 1). Elevating the pyrolysis temperature significantly increased the number of pores, specifically for biochars derived from eggplant.

### Acid neutralization

**Figure 2:**
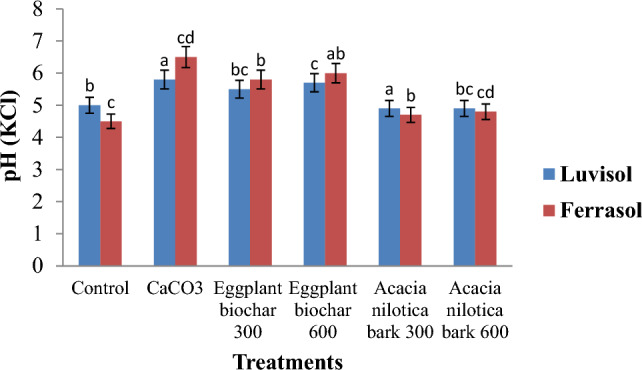
pH (KCl) units during 10-days incubation of soils from ferralsol and luvisol treated with CaCO_3_ = calcium carbonates and biochars from eggplant and *Acacia nilotica* bark created at different pyrolysis temperatures. Lime applied at recommended rate. Eggplant biochar at 300 °C; Eggplant biochar 600 °C and *Acacia nilotica* bark biochar at 300 °C; *Acacia nilotica* bark biochar 600 °C. The vertical error indicates LSD (*P* < 0.05).

The use of biochar resulted in a notable increase in soil pH, except for the Acacia nilotica bark biochars, which had no impact on the Luvisol soil (Fig. 2). In the case of Ferralsol, the incorporation of *Acacia nilotica* bark biochar resulted in soil pH levels that were comparable to the control, but lower than positive control (CaCO_3_). The application of eggplant biochar at 300 °C in Ferralsol yielded comparable results to the application of eggplant biochar at 600 °C in Luvisol. The incorporation of eggplant at a temperature of 300 °C in Ferralsol had a comparable impact to the positive control used in Luvisol. All biochar treatments exhibited a lower pH compared to the positive control across all soil types.

## Discussion

The biochar characterization reveals significant variations in materials composition derived from various feedstocks at the same pyrolysis temperatures^33^. Variations in the yield of biochar were also indicative of differences in the characteristics of the raw materials used. *Acacia nilotica* bark biochar, generated at temperatures of 300 °C and 600 °C, exhibited greater yield compared to eggplant residue-derived biochars. This can be attributed to the increased thermal stability resulting from the compact and rigid structure of lignin, as stated by Chen et al.^34^. This is similar to the findings of Diagboya et al.^35^ and Fardi et al.^36^, who observed a substantial yield of biochar from biomass materials rich in lignin. The pyrolysis of eggplant residue led to significant mass reduction, mainly attributed to the abundant cellulose content present in biomass^37^. The lower yield of eggplant biochar compared to *Acacia nilotica* bark biochar can be attributed to the evaporation of water vapor, thermal breakdown of lignocellulosic elements, and the loss of carbon as carbon monoxide. This matches other studies on the pyrolysis of biomass^38^ and is in line with the reduction of functional groups and the rearranging of carbon groups^39^.

The decrease in volatile matter observed at elevated pyrolysis temperatures (as shown in Table 3) can be due to the depletion of low molecular weight functional groups, including aliphatic molecules, which undergo aromatization as the temperature of pyrolysis increases. Karami^40^ observed similar patterns in the biochars derived from corn biomass. The eggplant biochars produced at a temperature of 600 °C exhibited a larger volatile matter compared to *Acacia nilotica* bark. This difference can be attributed to variations in the composition of lignin, hemicellulose, and cellulose between the two materials^41^. Torres-Sciancalepore et al.^42^ found that materials containing a significant amount of cellulose generate biochar with highly volatile matter. The presence of volatile matter has an impact on the stability of materials^43^, the availability of nitrogen, and ultimately the growth of plants^44^. The study conducted by Hanafi et al.^45^ found that biochars with a volatile matter content of 40% exhibited a breakdown rate of less than 10% over 100 years. The higher volatile matter content found in eggplant residue-derived biochar may serve as a valuable source of easily decomposable carbon (labile carbon) for microbial populations, as demonstrated by Vilas-Boas et al.^46^. However, this greater volatile matter content could potentially have adverse effects on carbon storage, as it may lead to a positive priming impact.

**Table 3:** Results of the yield and proximate analysis of the biochars.

Production temperature	Biomass type	Yield %	Volatile matter %	Moisture content	Fixed carbon %	Ash content %
300 °C	eggplant	32.8^d^	30.01^d^	4.26^c^	51.01^a^	20.31^e^
	*Acacia nilotica* bark	51.3^e^	40.03f.	2.67^ab^	57.9^c^	0.93^a^
600 °C	eggplant	23.9^b^	12.03^b^	4.39^c^	61.71^d^	27.24f.
	*Acacia nilotica* bark	34^d^	5.84^a^	1.81^a^	92.01f.	2.29^b^

*Values in the same column with distinct letters indicate statistically significant differences (*p* < 0.05). The letters "ab" signify that there is no significant difference between the mean values of groups labeled with the letter "a" and those labeled with the letter "b".

The rise in the level of ash as the pyrolysis temperature increases (Table 3) may be attributed to the inorganic substances accumulation, such as metals, silicate, potassium, calcium carbonates, and iron in the biochar^47^. Ash refers to the solid residue that is formed after the process of oxidizing all organic substances, such as carbon, hydrogen, and nitrogen^48^. The ash level of *Acacia nilotica* bark biochar is lower than that of eggplant biochar, which matches the findings of Boostani et al.^49^. Tang et al.^50^ conducted a comparative analysis between agricultural feedstock and wood-derived biochar. The ash content of biomass can be affected by the concentration of nutrients present^51^.

The rise in fixed carbon content as the pyrolysis temperature increases can be attributed to the reduction in volatile matter. These findings correspond with recent research on different forms of biochar^52^. The increased fixed carbon in *Acacia nilotica* bark biochars, in comparison to eggplant biochars, can be attributed to a greater lignin concentration. The presence of ash content serves as a fire-resistant element, hence impeding the breakdown of organic compounds and the creation of aromatic compounds^53^. This can elucidate the decreased fixed carbon content of eggplant biochar in comparison to *Acacia nilotica* bark. Similarly, Taksal et al.^54^ documented a negative correlation between the amount of ash present and the concentration of fixed carbon. Our findings correspond with the research conducted by Nik Pauzi et al.^39^, which states that biochars containing more than 35% fixed carbon should have ash content below 30%.

The rise in carbon content as the pyrolysis temperature increases (according to Table 4) could be attributed to the higher degree of polymerization, resulting in the formation of a condensed aromatic C structure^40^. Comparable findings were documented for biochars derived from Eulalia Grass^55^ and wood-derived biochars^56^. The depletion of hydrogen and oxygenated groups (Table 4) observed in our study provides more evidence for our findings, indicating the degradation of weaker bonds in the biochars^57^. Similar to Schlederer et al.^58^, the biochar derived from *Acacia nilotica* bark exhibited a more substantial enhancement in carbon composition compared to other types of biochar. This can be attributed to its aromatic substructure. Conversely, the eggplant biochar had a comparatively lower rise in carbon composition, which may be attributed to a higher presence of labile carbon. The production of biochars with high carbon content at higher temperatures can enhance carbon sequestration by their ability to withstand microbial degradation^59^. Soils that have a very low amount of organic matter could be improved by adding biochar, especially due to its high carbon concentration. This strategy has the potential to be advantageous for farmers since it offers a straightforward means of augmenting soil organic carbon. The utilization of *Acacia*-wood-derived bark biochar, formed at a temperature of 650 °C, has the potential to effectively capture and store carbon due to its substantial carbon content. It is suggested to apply an amount of 22 t ha^−1^ of biochar for optimal results.

The nitrogen content of eggplant biochars was higher when pyrolyzed at a temperature of 300 °C compared to *Acacia nilotica* bark. This can be attributed to the presence of nitrogen-containing heterocyclic compounds (as shown in Table 4) resulting from the higher nitrogen content in the initial material. Nevertheless, the elevated pyrolysis temperature of 600 °C resulted in a reduction of nitrogen content in eggplant biochars. This phenomenon can be attributed to the evaporation of NH_3_ and volatile molecules containing nitrogen^60^. According to the literature, the concentration of nitrogen typically decreases when the temperature is between 500 and 800 °C^61^. The application of eggplant residue to the soil can provide over 120kg of nitrogen per hectare when applied at a rate of 1% (kg N ha^−1^). This is equivalent to 381 units of eggplant feedstock, 50 units of *Acacia nilotica* bark feedstock, 413 units of eggplant biochar at 300 °C, 50 units of *Acacia nilotica* bark biochar at 300 °C, 292 units of eggplant biochar at 600 °C, and 42 units of *Acacia nilotica* bark biochar at 600 °C. According to Oraon et al.^11^, the bark biochar of *Acacia nilotica* did not exhibit any alterations in its nitrogen concentration.

**Table 4:** Ultimate analysis of eggplant and *Acacia nilotica* bark derived biochars.

Production temperature	Biomass type	Total C %	Total N %	O	H	C/N	O/C	H/C
300 °C	eggplant	61.9^a^	2.50^e^	13.8^c^	3.91^b^	24.7^a^	0.018^d^	0.005^bc^
	*Acacia nilotica* bark	71.31^d^	0.351^a^	25.8^e^	3.87^b^	203.1^d^	0.030f.	0.004^c^
600 °C	eggplant	64.9^b^	1.92^c^	6.25^a^	1.82^a^	33.8^b^	0.008^b^	0.002^a^
	*Acacia nilotica* bark	89.8^e^	0.376^a^	4.9^a^	2.28^a^	238.8^e^	0.004^a^	0.002^a^

The C/N ratio of the *Acacia nilotica* bark biochar was higher compared to the eggplant biochar, which matches recent findings^62^. The increased C/N ratio may result in intensified nitrogen immobilization via microorganisms within soil. This can happen as a result of recalcitrant carbon or the existence of heterocyclic carbon^61^. The O/C and H/C ratios reduced as the pyrolysis temperature increased, primarily due to the depletion of oxygen, hydrogen, and polar surface functional groups. Consequently, the carbon content increased^33^. The overall reduction in the levels of both oxygen (O) and hydrogen (H) indicates that *Acacia nilotica* bark and eggplant undergo similar condensation processes. All the biochars used in this work have H/C ratios below 0.7 and O/C ratios below 0.5, which are suitable for carbon sequestration^63^. The biochars created in this study, with an O/C range of 0.3 to 0.7 at 300 °C, are estimated to have a half-life of 100 to 1000 years. For biochars with an O/C ratio of less than 0.3, such as those generated at 600 °C in this work, a half-life exceeding 1000 years is proposed^63^.

Gezahegn et al.^64^ extended the idea proposed by Al-Swadi et al.^65^ by including the role of volatile materials in storing carbon. Volatile matter content beyond 80% indicates a lack of potential for carbon sequestration. Volatile matter content below 80%, along with an O: C ratio exceeding 0.2 or H: C ratio exceeding 0.4, suggests a moderate potential for carbon storage. Conversely, volatile matter content below 80%, combined with an O: C ratio below 0.2 or H: C ratio below 0.4, shows a strong potential for carbon sequestration. The lower O/C indicates the specific configuration of the aromatic rings, which enhances the stability of the biochar^1^. The increased O/C ratio observed at 600 °C for eggplant suggests a greater abundance of functional groups in the charcoal^61^. Processing residue into biochar is a feasible approach to sequester carbon and enhance soil organic C, which will endure in soils for an extended period.

The pH increased as the pyrolysis temperature increased, as shown in Table 5. This observation corresponds to the findings of Shao et al.^66^ and Ghorbani et al.^33^, who also showed an increase in alkaline biochars with higher pyrolysis temperatures. This phenomenon is linked to a rise in the concentration of salt in the ash content, as well as an increase in calcium carbonate and a reduction in acidic surface functional groups, resulting in the prevalence of oxygen functional groups^61^. The biochar derived from eggplant exhibited a notably greater capacity for liming compared to the biochar obtained from *Acacia nilotica* bark. The eggplant biochar exhibits greater values of carbon cation exchange capacity and pH compared to *Acacia nilotica,* as demonstrated by Oraon et al.^11^. Previous studies have shown a wide range of pH values, ranging from relatively acidic to very alkaline^64^, about the kind of raw material and pyrolysis temperature^61^. These findings are corroborated by the existence of carbonates and the resulting higher CCE. A study by Smider and Singh^67^ found similar results for tomato-derived biochar. Using biochars derived from eggplant in acidic soil resulted in an elevation of soil pH, whereas the use of *Acacia nilotica* biochars at equivalent levels of CCE had no impact on the pH level of the soil. The variations in the liming capacity of the treatments may arise from variances in the rate at which alkaline salts in the ash of the biochars dissolve^68^. Moreover, the process of dissolving certain alkaline salts in soils exceeds 10 days and exhibits variations. The rise in soil pH may be attributed to the existence of negatively charged functional groups in the eggplant biochars, which effectively bind hydrogen ions in the soil solution. The rise in soil pH after the addition of eggplant biochar may be attributed to its high pH and the ability of Ca^2+^ to displace Al^3+^ and H^+^ ions, resulting in the neutralization of hydrogen ions in the solution^69^. High alkalinity was identified as the cause for the similar outcomes observed in biochars derived from rice, as reported by Jiang et al.^70^.

**Table 5:** Chemical characteristics of biochar derived from woody (*Acacia nilotica* bark) and non-woody (eggplant) biomass.

Production temperature	Biomass	pH (KCl)	pH (H_2_O)	P (mg/kg^−1^)	K Cmol_c_ kg^−1^	Ca Cmol_c_ kg^−1^	Mg Cmol_c_ kg^−1^	CEC Cmol_c_ kg^−1^	CCE% Cmol_c_ kg^−1^
300 °C	eggplant	10.2^c^	10.8^c^	188^b^	12.1^b^	2.40^d^	7.40^c^	32^c^	10.14^ab^
	*Acacia nilotica* bark	4.69^a^	6.71^a^	0.0^a^	0.88^a^	0.59^a^	0.401^a^	4.10^a^	9.02^a^
600 °C	eggplant	11.9^e^	12.8f.	1232^e^	11.2^b^	3.50^a^	3.30^b^	11.3^b^	20.17^c^
	*Acacia nilotica* bark	9.09^b^	9.21^b^	0.0^a^	2.60^a^	1.69^c^	0.190^a^	2.91^a^	9.21^ab^

It is important to recognize that rectifying acidic-nature soils should not be based exclusively on the pH level. The liming property, which is influenced by the amount of ash present, should also be considered. Small-scale farmers are encountering difficulties in rehabilitating acidic soils, primarily due to the exorbitant expenses associated with lime. Therefore, use eggplant 600 °C biochar as a substitute could prove beneficial for small-scale farmers since it would be more cost-effective than limestone. Nevertheless, it is important to note that there may still be a need for additional lime because of the limited availability of biochar. Alleviating the acidity of the soil increases its pH, so enhancing the availability of nutrients, particularly phosphorus (P), and promoting microbial activity within the soil. Applying, it at a rate of 10 t ha^−1^ leads to insufficient reduction of soil acidity in acidic soils.

The high extractable phosphorus found in eggplant biochar can be attributed to its high ash content, which subsequently leads to an elevation of soil pH. Furthermore, it is important to note that phosphorus does not evaporate and dissipate into the atmosphere, especially at pyrolysis temperatures that are lower than 700 °C^59^. The Phosphorus that is present may be partially attached to –O– (phytic acid) in the carboxylic group. Another possible explanation is the precipitation of Phosphorus with Ca^2+^ to produce an apatite, a phenomenon frequently observed at elevated pyrolysis temperatures^57^. This is likely due to the alkaline nature of biochar and its abundance of carbonate ions. The presence of the phosphate functional group is observed exclusively at a temperature of 300 °C, but not at 600 °C, in eggplant biochar. Eggplant biochars can boost the availability of phosphorus (especially in acid soils) because of their liming properties, which is beneficial for agriculture. Application rates of phosphorus (kg P/ha) were estimated at a 1% rate of application for the feedstocks eggplant and *Acacia nilotica*. The biochars produced from these feedstocks through pyrolysis at 300 °C and 600 °C had corresponding application rates of 3.4 and 0-kg P/ha, and 19.3 and 0 kg P/ha, respectively. This was done under the assumption that phosphorus would not be held constant. Given this premise, it can be concluded that none of the materials would be adequate to achieve a phosphorus content of 60 kg to 100 kg per hectare. Utilizing biochar combined with chemical phosphorus fertilizers may serve as a viable alternative to substrates, as charcoal releases nutrients at a slower rate^71^. Feedstocks have a lesser amount of accessible phosphorus, which means that they will need to be applied at higher rates than their biochars. Therefore, converting biomass to biochar is a favorable choice.

The elevated concentrations of potassium (K) and magnesium (Mg) in the biochars derived from eggplant, shown in Table 5, match the findings of Diagboya et al.^35^. This study found that non-wood-based biochars generally exhibit higher levels of potassium and magnesium compared to biochars derived from wood. The reduction in magnesium (Mg) content as the pyrolysis temperature of eggplant increases can be attributed to the elevated presence of ash level at 600 °C. This suggests that the magnesium is likely in the form of carbonates, which cannot be extracted using ammonium acetate when compared to produce at 300 °C. This phenomenon is substantiated by the elevated pH observed at increased pyrolysis temperatures, which can be attributed to the discharge of hydroxide ions resulting from the reaction between carbonates and water^76^. The eggplant biochars have a much higher potassium (K) content compared to the *Acacia nilotica* bark. This difference may be attributed to the higher concentration of these elements in the respective feedstocks^56^. Nevertheless, the eggplant biomass had a higher potassium (K) concentration compared to its charcoal, indicating a gradual release of potassium during the pyrolysis process. Qiu et al.^72^ observed a decline in the concentration of potassium as the temperature of pyrolysis increased. This phenomenon can be elucidated by the binding of potassium to the carbonyl functional groups^73^ and the subsequent formation of a stable molecule (K_2_CO_3_)^37^, which render it unextractable via the ammonium acetate process. However, the feedstock's high concentration of available potassium does not make it an effective potassium supplement. This is because organic materials are vulnerable to nutrient loss when directly applied to the soil^74^. Henceforward, eggplant biochars have the potential to serve as an excellent soil additive for potassium and might potentially replace traditional sources of potassium. The cation exchange capacity values of biochar derived from non-woody feedstock are higher than those of biochar derived from woody biomass^75,77^. The present study revealed that *Acacia nilotica* bark exhibited a reduced cation exchange capacity in comparison to eggplant biochar. Increasing the temperature during pyrolysis resulted in a decrease in cation exchange capacity, potentially caused by the breakdown of organic volatile molecules and acidic functional groups that contribute to the negative surface charge of biochar^78^.

## Conclusion

Eggplant biochars exhibit lower fixed carbon and biochar yield while having high-volatile matter, nutrient content, and ash content in comparison to biochars derived from *Acacia nilotica* bark. Eggplant biochars exhibit elevated levels of potassium content, available phosphorus, pH levels, and calcium carbonate equivalent, particularly when exposed to higher pyrolysis temperatures. Adding eggplant biochar at a temperature of 600 °C raises the pH of the soil. This decrease in acidity leads to a decrease in the levels of aluminum and soluble iron, as well as the positive charges on aluminum and iron oxides. As a result, the fixation of phosphorus is reduced and its availability is increased in acidic soils. The pyrolysis of *Acacia nilotica* bark resulted in biochars with higher values of C/N, fixed carbon, and carbon content while exhibiting reduced levels of H/C, O/C, and nitrogen content, as the pyrolysis temperature increased. Biochars derived from the bark of *Acacia nilotica*, particularly those generated at a temperature of 600 °C, can capture carbon in the soil as a result of enhanced stability and aromatic properties. The results of this study validate the perspective that the kind of material used as feedstock is the main factor limiting the features of biochar. On the other hand, the temperature at which pyrolysis takes place functions as a modifier, affecting the chemical and physical characteristics of biochar and enhancing its aromatic quality. It is recommended to evaluate the agronomic potential of eggplant biochars in terms of their ability to provide potassium and enhance the availability of phosphorus in soils, considering their capacity to improve soil pH. Furthermore, it is necessary to comprehend the impact of incorporating these biochars into soils with near-neutral and acidic pH levels on factors such as pH, mineral nitrogen, CO_2_ emissions, available phosphorus, and potassium.

## Data Availability

All the raw data in this research can be obtained from the corresponding authors upon reasonable request.
